# Novel Approach in Biodegradation of Synthetic Thermoplastic Polymers: An Overview

**DOI:** 10.3390/polym14204271

**Published:** 2022-10-12

**Authors:** Raja Venkatesan, Madhappan Santhamoorthy, Krishnapandi Alagumalai, Rajesh Haldhar, Chaitany Jayprakash Raorane, Vinit Raj, Seong-Cheol Kim

**Affiliations:** School of Chemical Engineering, Yeungnam University, Gyeongsan 38541, Korea

**Keywords:** plastics, properties, biodegradation, pollution, environmental problems

## Abstract

Biodegradation is necessary for water-soluble or water-immiscible polymers because they eventually enter streams which can neither be recycled nor incinerated. It is important to consider the microbial degradation of natural and synthetic polymers in order to understand what is necessary for biodegradation and the mechanisms involved. Low/high-density polyethylene is a vital cause of environmental pollution. It occurs by choking the sewer line through mishandling, thus posing an everlasting ecological threat. Environmental pollution due to the unscrupulous consumption of synthetic polymers derived from petroleum has an adverse impact on the environment since the majority of plastics do not degrade, and the further incineration of synthetic plastics generates CO_2_ and dioxin. This requires understanding the interactions between materials and microorganisms and the biochemical changes involved. Widespread studies on the biodegradation of plastics have been carried out in order to overcome the environmental problems associated with synthetic plastic waste. Awareness of the waste problem and its impact on the environment has awakened new interest in the area of degradable polymers through microbes viz., bacteria, fungi, and actinomycetes. The microbial degradation of plastics is caused by certain enzymatic activities that lead to a chain cleavage of polymers into oligomers and monomers. This review focuses on the biodegradation rate of plastics by fungal and bacterial communities and the mode of action of biodegradation.

## 1. Introduction

**Figure 1:**
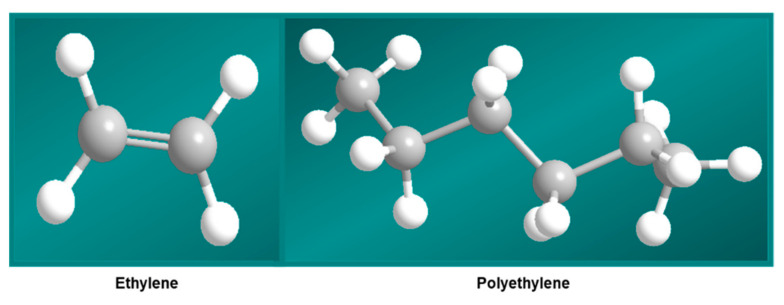
The 3D structures of ethylene and polyethylene.

The word plastic derives from the Greek word “*plastikos”* which means ”moldable into many shapes” [1]. Polyethylene (PE) is the most prevalent polymer in plastics, and it is formed from ethylene monomers (CH_2_=CH_2_). Figure 1 shows the 3D structure of ethylene and polyethylene. Polyethylene is not biodegradable in its natural state. India had 4.3 million tons of plastic demand in 2001–2002. That demand more than doubled by 2021–2022, climbing to 8.4 million tons per annum [2]. The plastic with the greatest demand in India in the year 2020 was polyethylene, at 33%. Polypropylene was second, with a 32% share of the demand [3]. Worldwide, the use of polyethylene is increasing at a rate of 12% per year, with the manufacturing of synthetic polymers reaching around 140 million tons per year [4]. With such a big amount of polyethylene accumulating in the environment, plastic-waste environmental issues arise [5]. These polymers are one of the most serious environmental hazards [6,7]. The most often used synthetic polymers are high-density (HDPE) and low-density polyethylene (LDPE) [8,9]. Plastics are disposed of, in many nations, through open, uncontrolled burning and landfilling [10,11]. Open burning emits toxins into the atmosphere, which can lead to a variety of health issues. Furthermore, burning plastics produces persistent organic pollutants, which have been linked to a number of negative impacts in humans. Municipal officials confirmed that carry bags are the major cause of clogged sewers as these prevent municipal solid waste from being burnt, which leads to an accumulation of waste, sludge, and rubbish. They discharge hazardous compounds into the environment, contaminating food [12,13]. The global production of plastic waste is estimated to be around 57 million tons per year [14]. To address the environmental issues related to synthetic plastic waste, numerous studies on the biodegradation of plastics have been conducted. Any physical or chemical change in the polymer as a result of environmental variables such as light, heat, moisture, chemical conditions, or biological activity is classified as the degradation of plastics. The term “biodegradation” describes the biological degradation of materials in natural environments, including synthetic polymers [15,16,17,18]. Fungi are commonly utilized in bioremediation because of their hardiness and abundance of enzymes. *Phanerochaete chrysosporium*, commonly known as white-rot fungus, is one of the most widely reported fungi, and its strong enzymes allow it to break down a wide spectrum of persistent pollutants and xenobiotics in nutrient-limited conditions. The capacity to breakdown low-density polyethylene (LDPE) used in widely available plastic carry bags was nearly 8% [19]. Since synthetic polymers are regarded as practically inert, efforts to isolate specific bacteria capable of exploiting them were discussed in this review [20,21].

## 2. Plastics

According to the American Society for Testing and Materials (ASTM) D6098-16, a plastic is any of a wide range of diverse materials that can be molded into useful shapes and designs that are completely or mainly organic in nature [22]. Whether individually or together, pressure and heat are applied.

### 2.1. Plastics and Their Properties

Plastics are polymers, or solid materials, that become flexible if heated and may thus be placed into molds. They are resistant to microbial attack and do not easily decompose in the environment [23,24] due to their excessive molecular mass, large number of aromatic rings, special bonds, or halogen substitutions. HDPE and LDPE are the synthetic polymers which are used the most often [8]. Because of its versatility and effectiveness, LDPE has been widely employed in this context. LDPE has more branching (on roughly 2% of the carbon atoms), weaker intermolecular interactions, lower tensile strength, and higher resilience. LDPE is still the slowest degrading sample [25,26]. Its obstinacy stems from its complicated three-dimensional structure and hydrophobic properties [27]. It possesses good resistance to aldehydes, ketones, and vegetable oils, as well as dilute and concentrated acids, alcohols, bases, and esters. As a result, they may survive for a long time in the environment without deteriorating [28].

### 2.2. Types of Plastics and Their Uses

Thermoplastics or thermosetting plastics are the two types of plastics [29]. The distinction between these two types of plastics is that thermoplastics can be melted and molded repeatedly, but thermosetting plastics can be heated and shaped only once and are, therefore, not generally recyclable. Polyurethane (PU) and polyester, including polyethylene terephthalate (PET), are two of the most common thermosetting plastics [30]. The most commonly used thermoplastic polymers are HDPE, LDPE, linear low-density polyethylene (LLDPE), polyvinyl chloride (PVC), polypropylene (PP), and polystyrene (PS). HDPE and LDPE are the most commonly used synthetic polymers [31]. Plastic films for carry bags, mugs, packaging, rubbish bags, and other applications are currently made from polyolefin-derived plastics such as polyethylene [32]. Food, pharmaceuticals, cosmetics, detergents, and chemicals all benefit from synthetic plastic packaging. The many types of polymers and their applications are listed in Table 1.

### 2.3. Production and Disposal of Plastics

Plastic is a flexible synthetic material formed from ethylene and a semi-crystalline high-molecular-weight polymer. Basic materials obtained from oil, coal, and natural gas are used to produce plastics [33,34,35,36,37]. Roughly 15.46 tons of plastic waste is processed in India every day, only 40% of which can be recycled, and the remaining 60% is impossible to dispose of, according to a 2003 survey. It is estimated that there has been 5.6 million tons of post-consumer plastic trash annually from the years 2008–2009 [38]. India uses 5.8 kg of plastic per person, which is a remarkably low level. The total amount of plastic used in India may have increased from 7.5 to 15.0 million tons by 2015, making it the third-largest consumer of plastics in the world. Around 12.75 million tons of plastics were required commercially in India in 2012. The amount of plastic waste has substantially increased due to the shortage of available landfill space [39]. The cost for the disposal of solid waste has increased as a result. Plastic pollution in the environment has become a major concern that can lead to long-term issues with the environment, the economy, and waste management. The term “white pollution” describes the grave threats posed by waste plastic in the environment [40,41]. Long-term plastic waste disposal in the soil decreases water penetration, reduces soil fertility, and limits plant growth, causing environmental issues [33]. The physical recycling of these materials has been shown to be inefficient and typically undesired [42] as biological molecules often infect plastic trash. The best option for the disposal of plastics seems to be utilizing plastics that can re-enter the biological life cycle through biodegradation [43].

### 2.4. Uses and Hazards of Plastics

Plastic materials have been used for a wide range of applications in recent years [44,45]. Plastic has become a part of modern life and is used in many different sectors. Plastics are extensively used in the packaging of products such as food, pharmaceuticals, cosmetics, detergents, and chemicals. Each year, millions of plastic bags adulterate the environment due to their improper disposal [46]. Plastic bags, being resistant to bacterial degradation, inevitably become a major threat to the environment despite their commercial success. A plastic bag takes an average of one thousand years to decompose completely. During this time, it breaks into small pieces which remain embedded in the soil. The possibility of the material breaking down and mixing with the soil increases with the thickness, which adversely affects both marine life and the soil itself [47]. Those that emit harmful chemicals contaminate food [48]. Plastic waste threatens a lot of hazards to marine life due to its buoyancy, long-term persistence, and ubiquity in the marine environment [49]. In many countries, plastic waste is disposed of through landfilling and uncontrolled, open burning [50]. Open pruning emits air contaminants which may contribute to a number of health issues. Chemicals used in the manufacturing of vinyl, such as ethylene dichloride and vinyl chloride, are believed to cause cancer. They may also cause damage to the liver, kidneys, or nervous system, among many other health issues. One of the most significant and persistent changes in the environment is the development of fragmented polymers, or PE [51,52]. Since the start of industrial production, plastic pollution has accumulated in both terrestrial and marine environments in just a few decades [53,54]. Many marine animals may eat these particles by mistaking them for plankton. As a result, it is possible that ingested plastic debris will enter and accumulate in the food chain, exerting multiple hazards [55]. With the amount of polyethylene which has accumulated in the environment, it will take thousands of years for the polyethylene to degrade completely [56]. The world faces a major problem when it comes to the use of plastics, most notably those used for packaging [57,58].

## 3. Biodegradation

**Figure 2:**
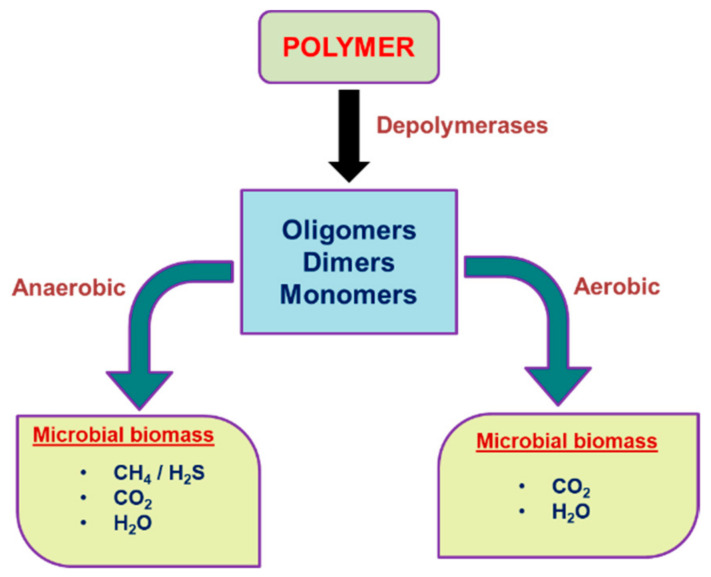
Scheme of polymer degradation under aerobic and anaerobic conditions.

Biodegradation is the deformation of a substance into new compounds through biochemical reactions or the actions of microorganisms such as bacteria or fungi; alternatively, biodegradation is the process by which microbial organisms transform to alter (through metabolic or enzymatic action) the structure of chemicals introduced into the environment [59,60,61,62]. Figure 2 shows polymer degradation under aerobic and anaerobic conditions.

### 3.1. Biodegradation of Plastic by Microbes

Biodegradation is a natural and complex process of decomposition facilitated by biochemical mechanisms and the successive mineralization of the polymer material. Biodegradable plastics open the way for new waste management strategies since these materials are designed to degrade under environmental conditions or in municipal and industrial biological waste treatment facilities [63]. Bacteria, fungi, and actinomycetes are of particular interest in the biodegradation of natural and synthetic polymers. Many species or types of microorganisms are found broadly in nature [64]. Microorganisms are highly adaptive to their environment and secrete both endoenzymes and exoenzymes that attack the substrate and cleave the molecular chains into segments [65]. The biological degradation of these polyethylene films has been reported in pure culture studies with various microorganisms such as *Staphylococcus* sp., *Streptococcus* sp. [66], *Phanerochaete* sp. [67], and *Bacillus* sp. [68]. Mergaert et al. investigated the polymer-degrading isolates [69], actinomycetes, and fungal isolates which were able to degrade polyhydroxy butyrate, bionolle, and polycaprolactone. Orhan et al. investigated the PE biodegradation process by using fungal isolates [70], *Phanerochaete chrysosporium,* and their extracellular polymers such as polysaccharides which can help to colonize the polymer surface [71]. Clutario et al. showed the physical evidence of the colonization of polyethylene strips by *Xylaria* sp. isolated from a termite comb. *Xylaria* sp. can utilize polyethylene plastic as a co-carbon source, thereby degrading them into usable forms for self-substance [72]. Saminathan et al. isolated a strain identified as *P. putida* by performing appropriate degradation on disposable plastic items [73]. Thilagavathy et al. reported that *Xylaria* sp. from the fungal garden variety of termite could degrade 20 µm thicknesses of LDPE plastics to 25% in a period of 50 days of incubation at 25 °C [74]. Though the fungi survived in the environments with poor nutrient supply, pH, and moisture availability, they could degrade the plastic successfully to the appreciable value. The faster growth of fungal biomass compared to bacteria, the growth extension, and the penetration into other locations in the plastic are possible through the distribution of hyphae. The distribution and penetration ability of their fungal hyphae was an added advantage [29].

### 3.2. Mechanism of Biodegradation

**Figure 3:**
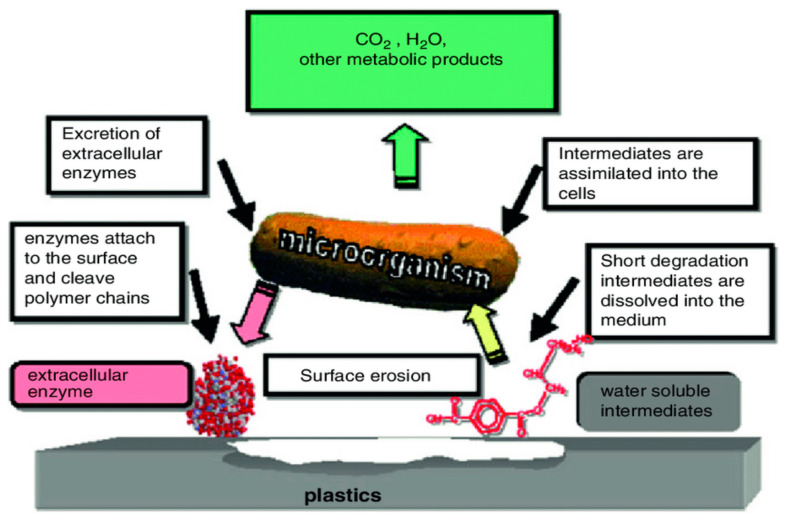
General mechanism of plastic biodegradation under aerobic conditions, adopted from [88] with permission from Elsevier through copyright clearance center.

**Figure 4:**
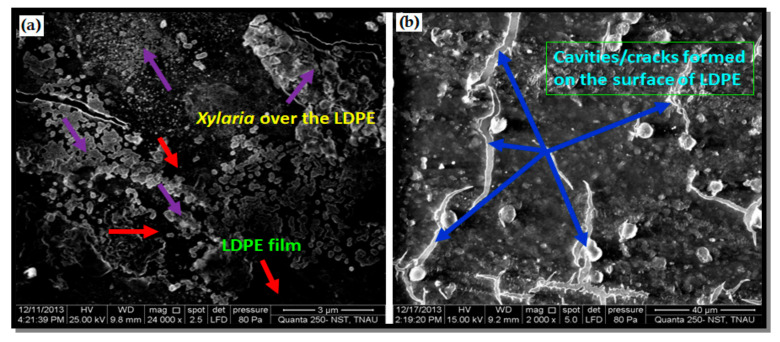
(**a**) SEM images of Xylaria over the LDPE, (**b**) cavities/cracks formed on the surface of LDPE. The LDPE surface is shown by the red color arrows. The Xylaria is depicted by the violet-colored arrows above the LDPE.

The general mechanism of plastic biodegradation is shown in Figure 3. In biological force, the growth of many fungi can cause small-scale swelling and bursting, as the fungi penetrate the polymer solids [75]. Plastics are potential substrates for heterotrophic microorganisms [76]. The microbes excrete extracellular enzymes which depolymerize the polymers outside the cells. Physical forces, such as heating, cooling, freezing, and drying can cause mechanical damage such as the cracking of polymeric materials [77]. When exposed to soil, natural polymers such as starch, cellulose, and proteins are destroyed by a microbiological process [78]. The microbial colonization of a polymer surface is the first requirement for its biodegradation [79]. During degradation, exoenzymes from microorganisms break down complex polymers, yielding smaller molecules of short chains, e.g., oligomers, dimers, and monomers that are small enough to pass the semi-permeable outer bacterial membranes and then be utilized as carbon and energy sources. The process is called depolymerization. The end products are CO_2_, H_2_O, and CH_4_, and the degradation is called mineralization [80,81]. To facilitate the biodegradation of these polymers, a preliminary step of photo-oxidation or thermo-oxidation has routinely been employed. This oxidation of the polymer results in the formation of carbonyl residues that can be consumed by non-specific microbial populations [82]. The results change in the bond scission, chemical transformation, and formation of new functional groups [83]. Very small variation in the chemical structure of polymer could lead to large changes in their biodegradability. Scanning electron microscopy (SEM) is a useful imaging approach for the visualization of different polymers because it provides a consistent picture of the polymer morphology as a non-uniform structure characterized by variable thickness and variable polymer density. Akutsu et al. illustrated the surface topography of polymers with high resolution [84]. The morphological changes in PUR during the enzyme reaction were observed by scanning electron microscopy. Ikada et al. obtained information about the degradation mechanism; the observations can be made using either scanning electron microscopy (SEM) or atomic force microscopy (AFM) [85]. Clutario et al. observed the mycelia of fungus on polythene after sufficient adaption to the lab condition [73]; the *Xylaria* fungus penetrated into the plastic material of LDPE. After that, some chemical reaction must have taken place since evidence of bio-corrosion by the mycelium was observed through the SEM, including the tearing, pitting, and striating of the plastic strip incubated with fungus. Physico-mechanical properties have also been determined before and after the degradation of film in order to understand the rate as well as the mechanism of degradation [86,87]. SEM images of *Xylaria* over the LDPE film are shown in Figure 4.

### 3.3. Enzymatic Degradation of Plastic

The biodegradation of polymers is catalyzed by extracellular, degradative enzymes that produce water-soluble, low-molecular-weight products from the macromolecular substrates. These products are water-soluble and can diffuse into the surrounding aqueous environment to be taken up by the cells of the microorganisms and used as nutrients. Mergaert et al. reported various natural polyesters, such as polyhydroxybutirate and polycaprolactone, that can also be degraded and assimilated by various microbial populations [70]. Webb et al. have studied how the biodegradation of polythene begins with the attachment of microbes in the surface of the polymer [89]. The microbes such as bacteria (*Streptomyces viridosporus*T7A, *Streptomyces badius*252, and *Streptomyces setonii*75Vi2) and wood-degrading fungi produced some extracellular enzymes which led to the degradation of polythene. Polythene-cleaving enzymes belong to the group of hydrolases, which catalyze the hydrolytic cleavage of the C-O and C-N- bonds. Hydrolases include lipase and esterase enzymes. Tokiwa et al. revealed that the various esterases and lipases produced were hydrolyzing the PCL.

**Figure 5:**
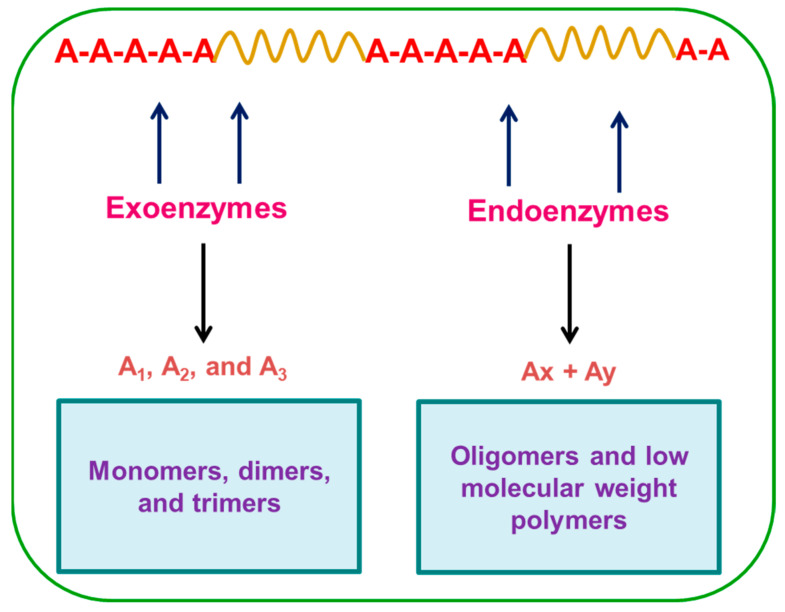
Enzyme-catalyzed degradation processes.

**Figure 6:**
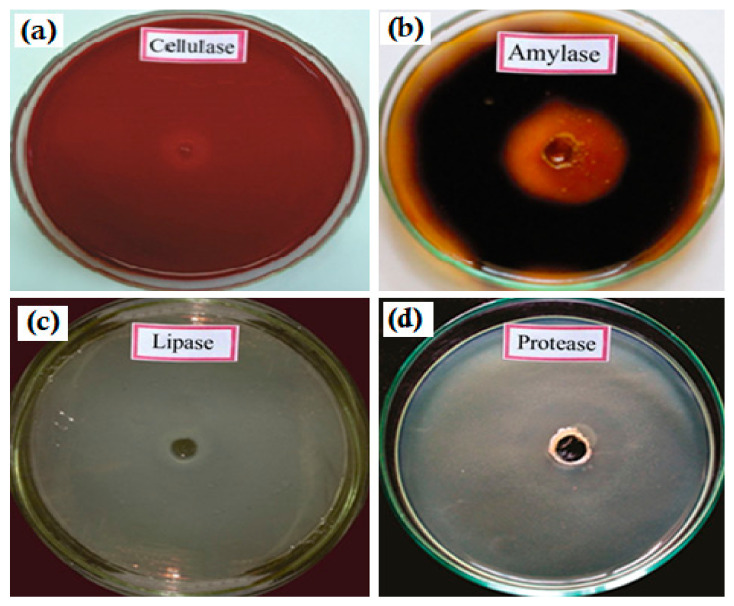
Hydrolysis activity of Xylaria sp in LDPE. Secretion of depolymerise in the GYP medium by Xylaria sp (xtc 1) when grown on media containing (**a**) cellulase; (**b**) caesinase; (**c**) lipase and (**d**) gelatinase respectively, as seen by the zones of clearing.

Particularly, the lipases of *Rhizopusdelemar* and *R. arrhizus* were used in the hydrolysis of polymers. Thilagavathy et al. identified that *Xylaria* sp. of termite fungal comb was able to degrade 20 µm of thickness of LDPE plastics through their secretion of depolymerizers (Figure 5) [74]. The oxidized polymer helps in the adhesion of microorganisms (due to probable changes in the hydrophobicity of the polymer surface), which is a prerequisite for biodegradation. Johnson et al. investigated how the microorganism growing on plastic material may either utilize the plasticizer molecule of starch cellulose or the other polymer [90]. Fungi are able to degrade a wide variety of polymers through the production of several enzymes such as cellulase and amylase [91,92,93]. The active enzymes have been grouped as esterases, lipases, proteases, and ureases which degrade the polyurethane substrate by cleaving the ester bonds [94]. The enzyme reacts with solid polyester PU to hydrolyze the ester bounds of PU (Table 2). The hydrolysis of esters bound in PU is postulated to be a mechanism of PU biodegradation (Figure 6). Prema et al. studied and purified enzyme protease that was involved in the degradation of PLA [95]. Biological processes by both microbial and enzymatic activities are currently considered to be sustainable recycling methods in the biodegradation of plastics.

## 4. Future Prospects

Use of biodegradable polymers in specific applications, such as packaging, agriculture, and the health industry, is the most innovative and environmentally safe way to deal with issues related to the disposal of plastic waste produced from different sources. If it is used, bio- and fossil-based biodegradable polymers efficiently decompose in the environment, in cells, or in well-maintained industrial applications. The environment is currently highly affected by non-biodegradable petrochemical materials used in the manufacture of plastics, particularly in the absence of waste disposal facilities and littering controls. In certain applications, the demand for eco-friendly polymers is still increasing. Future progress should concentrate on the use of these materials, especially for the manufacture of packaging materials, food item packaging, and disposable medical aid. Introducing biodegradable plastics in agricultural film, fishing nets, pharmaceuticals, surgical frameworks, and sterile goods is also beneficial for the environment. Furthermore, it is suggested to use biodegradable plastics in scenarios in which there is a great risk of diffusion into the environment or when it is difficult to distinguish the waste. However, in order to profit from certain polymers in the community, efficient waste management and littering avoidance should be in action. For specific application, the new generation of bio-based biodegradable plastics will promise to foster a more environmentally friendly society. In addition, for these plastics to be reusable afterwards, materials should also be biodegraded and recycled in a balanced way. For this, it is essential to have a thorough understanding of the structure of biomass production in nature in order to manufacture new biodegradable plastic polymers by making small structural reforms. Organisms, synthetic scientists, process engineers, and bioenergy researchers should all collaborate in order to develop environmentally friendly goods that will increase society’s sustainability.

## 5. Conclusions

This review has covered the major concerns about the synthetic polymers, their types, uses, and degradability. It has looked at the disposal method and the standards used in evaluating polymer degradation. The biodegradation of plastic is an innovative means of solving the plastic disposal problem from the standpoint of developing new techniques. Based on the literature survey, it can be concluded that polythene is very useful in our day-to-day life to meet our desired needs. It can be used to package goods, food, medicine, scientific instruments, and other materials. Its use is increasing daily as a result of its exceptional value. However, one of the greatest hazards to the environment is plastic. Two main efforts have usually been explored to minimize the environmental impact of plastic waste: one is to manufacture biodegradable plastics, and the other is to isolate specific microorganisms to biodegrade plastic wastes. Microorganisms, especially bacteria or fungi, play a crucial role in biological degradation of polymers. Biodegradation causing various types of structural and chemical changes in the polymer has to be reduced. To handle the problems of plastic waste, the enzymatic biodegradation of plastic will enhance the biodegradation rate. In the near future, these microorganisms can be used to reduce the quantity of solid waste, which is rapidly accumulating in the natural environment.

## Figures and Tables

**Table 1:** Main plastics and their applications.

Plastics	Applications
LDPE, LLDPE, and PVC	Films and Packaging
PET, PVC, and HDPE	Bottles, Tubes, Pipes, and Insulation Molding
PS, PP, and PVC	Tanks, Jugs, and Containers
LDPE and LLDPE	Bags
PU	Coating, Insulation, Paints, and Packing

**Table 2:** Enzymatic activity of *Xylaria* isolates in biodegradation plastics.

Isolates	Enzyme Activity (IUml-1)			
	40th DAI			
	Cellulase	Amylase	Lipase	Protease
Xtc1	0.54 ± 0.02	0.27 ± 0.01	2.91 ± 0.35	1.28 ± 0.12
Xtc4	0.46 ± 0.04	0.15 ± 0.02	2.84 ± 0.41	0.89 ± 0.10
Xtc8	0.34 ± 0.03	0.23 ± 0.01	1.75 ± 0.20	0.76 ± 0.08
Xtc12	0.42 ± 0.02	0.17 ± 0.02	2.80 ± 0.29	1.01 ± 0.13
Xtc20	0.39 ± 0.03	0.25 ± 0.03	2.37 ± 0.31	0.99 ± 0.10
MTCC 3669	0.48 ± 0.02	0.31 ± 0.02	2.78 ± 0.25	1.14 ± 0.12

## Data Availability

Not applicable.
